# Computed Tomography Doses Calculation: Do We Really Need a New Dose Assessment Tool?

**DOI:** 10.3390/jcm14041348

**Published:** 2025-02-18

**Authors:** Arkadiusz Szarmach, Dominika Sabiniewicz-Ziajka, Małgorzata Grzywińska, Paweł Gać, Maciej Piskunowicz, Magdalena Wszędybył-Winklewska

**Affiliations:** 1 2nd Department of Radiology, Medical University of Gdansk, 80-210 Gdansk, Poland; dominika.sabiniewicz@gumed.edu.pl; 2 Neuroinformatics and Artificial Intelligence Laboratory, Department of Neurophysiology, Neuropsychology and Neuroinformatics, Medical University of Gdansk, 80-210 Gdansk, Poland; malgorzata.grzywinska@gumed.edu.pl (M.G.); magdalena.wszedybyl-winklewska@gumed.edu.pl (M.W.-W.); 3 Centre for Diagnostic Imaging, 4th Military Hospital, Weigla 5, 50-981 Wroclaw, Poland; pawelgac@interia.pl; 4 Department of Population Health, Division of Environmental Health and Occupational Medicine, Wroclaw Medical University, Mikulicza-Radeckiego 7, 50-368 Wroclaw, Poland; 5 1st Department of Radiology, Medical University of Gdansk, 80-210 Gdansk, Poland; maciej.piskunowicz@gumed.edu.pl; 6 Institute of Health Sciences, Pomeranian University in Slupsk, 76-200 Slupsk, Poland

**Keywords:** computed tomography, radiation dose calculation, dose–length product, size-specific dose–length product, size-specific dose estimate

## Abstract

**Background/Objectives:** The increasing use of computed tomography (CT) scans significantly contributes to population exposure to ionizing radiation. Traditional dose metrics, such as dose–length product (DLP) and effective dose (ED), lack precision in reflecting individual radiation exposure. This study introduces a novel parameters such as size-specific effective dose (EDss) and the size-specific dose–length product (DLPss), to improve patient-specific dose estimation. The aim of this study is to enhance dose calculation accuracy, optimize CT protocols, and guide the development of next-generation CT technologies. **Methods:** A retrospective analysis of 247 abdominal and pelvic CT scans (113 women, 134 men) was conducted. Anthropometric parameters, including body mass index (BMI), cross-sectional dimensions, and dose indices, were measured. EDss and DLPss were calculated using size-specific correction factors, and statistical correlations between these parameters were assessed. **Results:** The mean BMI was 25.92 ± 5.34. DLPss values ranged from 261.63 to 1217.70 mGy·cm (mean: 627.83 ± 145.32) and were roughly 21% higher than traditional DLP values, with men showing slightly higher mean values than women. EDss values ranged from 6.65 to 15.45 mSv (mean: 9.42 ± 2.18 mSv), approximately 22% higher than traditional ED values, demonstrating improved individualization. Significant correlations were observed between BMI and effective diameter (r = 0.78), with stronger correlations in men (r = 0.85). The mean CTDIvol was 11.37 ± 3.50 mGy, and SSDE averaged 13.91 ± 2.39 mGy. Scan length reductions were observed in 53.8% of cases, with statistically significant differences by gender. **Conclusions:** EDss and DLPss offer improved accuracy in radiation dose estimation, addressing the limitations of traditional methods. Their adoption into clinical protocols, supported by AI-driven automation, could optimize diagnostic safety and significantly reduce radiation risk for patients. Further multicenter studies and technological advancements are recommended to validate these metrics and facilitate their integration into daily practice.

## 1. Introduction

It is estimated that the number of computed tomography (CT) scans performed worldwide increases by 3% to 4% annually [1]. This consistent growth in the number of scans, combined with the high effective dose (ED) associated with CT, makes it the most significant contributor, in percentage terms, to the total population’s exposure to ionizing radiation from diagnostic imaging [2,3,4]. Moreover, CT examinations are often repeated multiple times, leading to a cumulative radiation dose that may approach levels at which adverse effects on the body are observed. According to various sources, exposure to the harmful effects of ionizing radiation from diagnostic imaging increased by approximately 600% between 1980 and 2006 [5,6,7,8,9].

The risk of developing organ-specific cancers increases with the growing number of diagnostic examinations. It is estimated that up to 2% of such cancers may be directly caused by ionizing radiation used during CT scans [3]. Furthermore, the probability of stochastic radiation effects rises by 5% for every 1 Sv of ionizing radiation. For example, a single CT scan with an ED of 10 mSv (the reference dose for a CT scan of the chest and abdomen) increases the likelihood of such effects by 0.05%. This means that for every 10,000 patients examined, as many as 5 may develop some type of cancer as a result of radiation exposure [10]. Although the risk to an individual patient is not high, the increasing number of diagnostic examinations, including repeated scans, may lead to a rise in the absolute number of cancers within the population as a whole.

Commonly applied methods for estimating radiation doses, such as dose–length product (DLP), ED, and size-specific dose estimate (SSDE), are subject to certain methodological errors, meaning that they do not fully reflect the actual radiation risk [11,12,13]. A commonly used method for estimating doses in patients undergoing CT examinations involves measuring organ doses with standardized phantoms, which can be either physical or virtual. Physical phantoms are typically based on reference models with diameters of 16 cm or 32 cm, constructed from polymethyl methacrylate (PMMA) and commonly referred to as “head” or “body” phantoms. In contrast, virtual phantoms are designed to represent the 10th, 50th, and 90th percentiles of standing height and body weight. Unfortunately, these methods are time-consuming and require complex mathematical calculations based on a substantial amount of data [14,15,16,17].

Considering both the continuous increase in the number of CT examinations and their frequent repetition, it is crucial to develop reliable and precise methods for calculating and monitoring radiation doses. This will enable efforts to reduce radiation risk, optimize examination protocols, and modernize future generations of scanners.

This study was performed to analyze radiation doses dependent on selected anthropometric parameters of patients during CT examinations of the abdomen and pelvis. A new parameter, the size-specific ED (EDss), is proposed, which more accurately describes the actual radiation dose for individual patients. Additionally, an evaluation was conducted of a dose index introduced in a previous study (focused on chest CT), referred to as the size-specific DLP (DLPss), which estimates radiation risk [18].

## 2. Materials and Methods

With the use of dedicated software, MedStream Designer (Transition Technologies Science Ltd., Warsaw, Poland, 2015), a local database of abdominal and pelvic CT examinations performed between 2020 and 2022 was searched, yielding 3267 records.

The following exclusion criteria were applied: cachexia, significant obesity, history of major surgical procedures in the scanned area (e.g., colectomy, hepatectomy), advanced neoplastic disease or inflammation within the abdominal and pelvic organs, ascites, presence of motion artifacts or artifacts caused by foreign bodies, and low-quality imaging.

The inclusion criteria for the study were non-contrast abdominal and pelvic CT scans performed on adults with a body mass index (BMI) ranging from 18 to 35 kg/m^2^. In total, 247 scans were included in the final analysis (113 women and 134 men) (Table 1).

All examinations were performed using the SOMATOM Definition Flash scanner (Siemens, Erlangen, Germany). The age, body weight, and height of patients at the time of the examination were obtained from the header data included in the study records. The BMI was calculated for each patient using the standard formula [19]:

**BMI = weight (kg)/height^2^ (m)** (1)

The anteroposterior (AP) and lateral (LAT) dimensions of each patient were measured manually using an electronic caliper on the largest transverse scan. The effective diameter (Deff) was then calculated from the obtained data using the following formula:

(2) $$\mathrm{Deff}=\sqrt{AP\times LAT}$$

For each scan, standard information from the X-ray radiation dose report was obtained as included in the examination protocol. These data encompassed details such as tube voltage and current, the diameter of the reference phantom used, and radiation parameters, including the CT dose index volume (CTDIvol), DLP, topogram length, and the actual scanning range.

The SSDE was calculated by multiplying the CTDIvol value by a conversion factor ***k*** corresponding to each calculated Deff, based on the tables published in AAPM Report No. 204 [20].

**SSDE = CTDI_vol_ × *k*** (3)

The ED was calculated as the product of DLP and the conversion factor ***ƒ***, which, according to the data in AAPM Report No. 96 [21], is 0.015 mSv/mGy·cm for abdominal CT examinations:

**ED = DLP × *ƒ*** (4)

As previously mentioned [18], to calculate the DLP value specific to each patient, the parameter DLPss was introduced, expressed as the product of DLP and a conversion factor ***k*** dependent on the calculated Deff (obtained from the tables in AAPM Report No. 204) [20]:

**DLP_ss_ = DLP × *k*** (5)

For a more precise assessment of the ED received by the patient during abdominal and pelvic scanning, a new index called the EDss was proposed. This is the product of DLPss and the conversion factor ***ƒ*** obtained from AAPM Report No. 96 [21], according to the following formula:

**ED_ss_ = DLPss × *ƒ*** (6)

All calculations were performed using the statistical software Statistica version 13 (TIBCO Software Inc., Palo Alto, CA, USA, 2017). For continuous variables, the Shapiro–Wilk test was used to assess the normality of the distribution. For comparisons of independent groups based on sex and the anatomical area under study, Student’s *t*-test was applied for variables with a normal distribution, and the Mann–Whitney U test was applied for variables deviating from normality. Linear correlation coefficients were calculated using non-parametric statistics with Spearman’s rank correlation because most variables did not follow a normal distribution. Analyses were performed with a 95% confidence interval, and a statistical significance level of *p* < 0.05 was adopted for the hypotheses tested.

## 3. Results

### 3.1. Scan Length

In the study group, the average diagnostic scan length was shorter than the topogram scan length (Table 2). A reduction in diagnostic scan length relative to the topogram scan was observed in 53.8% of cases, while an increase occurred in 45.7% of scans.

Both the absolute and percentage differences between the diagnostic and topogram scan lengths were significantly greater in women than in men (*p* < 0.05).

### 3.2. LAT and AP Dimensions and Deff

The average LAT, AP, and Deff measurements were 350.30 ± 4.60 mm, 240.63 ± 4.43 mm, and 290.44 ± 4.32 mm, respectively (Table 3). For both sexes, none of the evaluated dimensions showed a statistically significant difference (*p* > 0.05).

**Figure 1:**
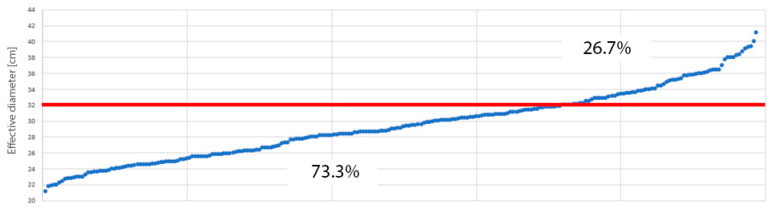
Percentage distribution of effective diameter in patients compared with reference phantom diameter for abdominal CT examination.

For the purposes of this study, the number and percentage of patients whose Deff deviated from 320 mm (the diameter of the reference phantom for abdominal CT) were assessed. In 73.3% of examinations, the Deff was smaller than 320 mm, while in 26.7% of patients, it exceeded this value (Figure 1).

### 3.3. Correlation of Deff with BMI

**Figure 2:**
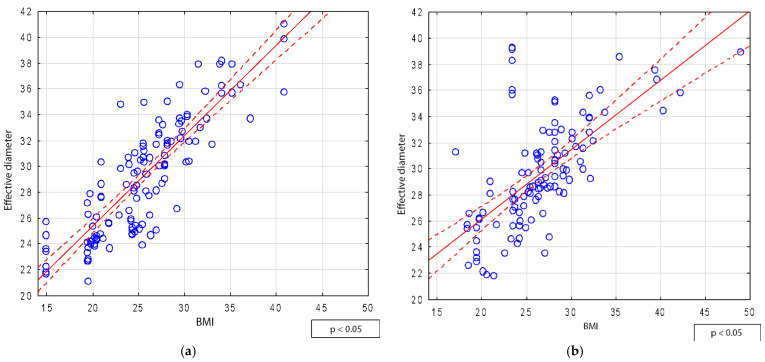
Relationship between BMI and effective diameter according to sex: (**a**) male and (**b**) female.

A significant correlation was observed between BMI and abdominal Deff for the entire study group, with r = 0.78. The correlation was stronger in men (r = 0.85) (Figure 2a) than in women (r = 0.68) (Figure 2b).

### 3.4. CTDIvol and Correlation with Selected Anthropometric Parameters

The CTDIvol value for abdominal and pelvic CT examinations in the entire group ranged from 5.86 to 29.51 mGy (mean: 11.37 ± 3.50 mGy). For women, it ranged from 6.98 to 24.56 mGy (mean: 11.61 ± 3.37 mGy), while for men, it ranged from 5.86 to 29.51 mGy (mean: 11.17 ± 3.61 mGy). The differences in CTDIvol values between sexes were not statistically significant (*p* = 0.13).

The study also analyzed the relationship between CTDIvol and selected anthropometric parameters of the patients. In women, the highest correlation coefficient was observed for the LAT dimension of the maximum cross-sectional area (r = 0.94), while in men, the strongest correlation was found for Deff (r = 0.92) (Table 4).

### 3.5. SSDE and Correlation with BMI

Based on Formula number (3) (see Section 2), the mean SSDE value in the study group was calculated to be 13.91 ± 2.39 mGy (range: 8.61–28.03 mGy). For women, the mean SSDE value was 14.14 ± 2.32 mGy (range: 10.47–23.33 mGy), while for men, it was 13.72 ± 2.44 mGy (range: 8.61–28.03 mGy).

**Figure 3:**
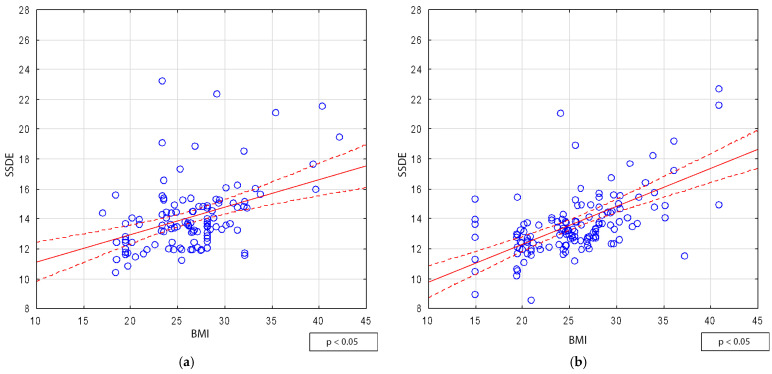
Correlation between SSDE and BMI: (**a**) female and (**b**) male.

A statistically significant but weak correlation between SSDE and BMI was observed in women (r = 0.38), whereas a stronger correlation (r = 0.55) was found in men (Figure 3a,b).

### 3.6. DLP and DLPss

The DLP values for abdominal CT in the entire group ranged from 165 to 1230 mGy·cm (mean: 514.88 ± 181.83). For women, the values ranged from 283 to 1189 mGy·cm (mean: 508.35 ± 171.73), while for men, they ranged from 165 to 1230 mGy·cm (mean: 520.38 ± 190.41). The differences between the sexes were not statistically significant (*p* = 0.69).

For the purposes of this study, a new parameter, DLPss, was introduced to assess the total radiation dose received by the patient, taking into account both the actual scan length and cross-sectional dimensions (see Formula number (5) in the Section 2).

The DLPss values for the entire study group ranged from 261.63 to 1217.70 mGy·cm (mean: 627.83 ± 145.32). For women, the values ranged from 343.44 to 1147.08 mGy·cm (mean: 617.47 ± 136.87), and for men, from 261.63 to 1217.70 mGy·cm (mean: 636.57 ± 152.03). The differences between women and men were not statistically significant (*p* = 0.166).

### 3.7. ED and EDss

The mean ED for abdominal CT in the entire group was 7.72 ± 2.73 mSv, with 7.62 ± 2.58 mSv for women and 7.81 ± 2.86 mSv for men.

To calculate the ED for a specific patient considering their actual dimensions, a new index (EDss) was proposed according to Formula number (6) (see Section 2).

**Figure 4:**
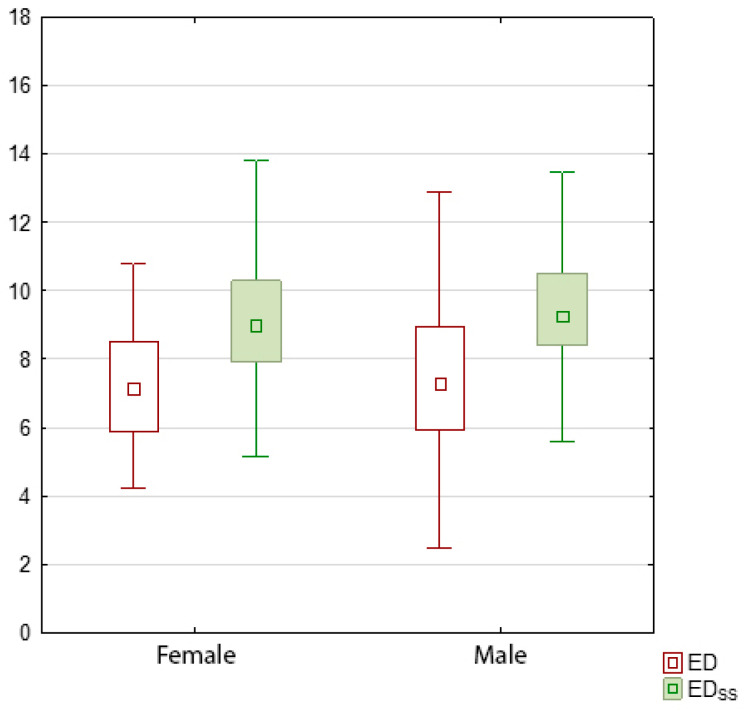
Correlation between ED and EDss in women (white) and men (green).

The mean EDss values in the study group were 9.42 ± 2.18 mSv, with 9.25 ± 2.06 mSv for women and 9.55 ± 2.28 mSv for men. The calculated EDss values were higher than the ED (Figure 4).

No statistically significant differences were found between ED and EDss based on sex (*p* = 0.69 and *p* = 0.16, respectively).

### 3.8. Evaluation of DLPss for Chest CT vs. Abdominal and Pelvic CT

Our analyses showed that the DLPss values for abdominal CT examinations were higher than those for chest CT (Table 5). For chest CT, statistically significant differences in the proposed parameter were observed between women and men (*p* < 0.05) [18]. However, in abdominal CT, no such relationship was found (*p* = 0.166).

### 3.9. Evaluation of EDss for Chest CT vs. Abdominal and Pelvic CT

The calculated, modified EDss values showed statistically significant differences between the sexes, but only for chest CT. By contrast, no statistically significant difference in this parameter was observed between women and men for abdominal CT (Table 6).

According to the calculations, the EDss values were higher than the traditionally calculated ED. For chest CT, the difference was approximately 19% (23% for women and 16% for men), while for abdominal CT, the difference was 22% (21% for women and 23% for men).

## 4. Discussion

The ionizing radiation dose received by a patient (patient ED) during a CT examination depends on several individual factors, including the scanner settings, choice of examination protocol, and type of scanner used. Additionally, imaging quality is closely linked to patient size. The contrast-to-noise ratio decreases as patient size decreases. Conversely, as the size of the scanned object increases, imaging quality deteriorates [22]. Therefore, using a standardized examination protocol for different patients is ineffective because it results in larger patients receiving images of poorer quality, while smaller patients are at risk of excessive radiation exposure.

Each CT examination begins with the acquisition of a topogram, the primary purpose of which is to determine the actual scanning range. Based on this, the radiographer adjusts the length of the examination area. This parameter is directly influenced by the patient’s height and clinical indications, while ensuring adherence to the ALARA/ALARP principle [23].

One of the most common causes of unnecessary additional radiation exposure is the expansion of the scanning area beyond the established anatomical boundaries of the body parts being examined [24,25,26,27,28]. Karla et al. [26] analyzed 106 CT scans of the abdomen and pelvis and found that 97% of the scans began above the dome of the diaphragm, while 94% included additional images extending below the pubic symphysis. On average, each scan included 12 extra images, increasing the scanning area by 60 mm, with most of these additional images providing no significant clinical information. This led to a 13.1% increase in DLP in studies conducted with fixed tube current and a 16.7% increase in scans using automatic z-axis current modulation. Similarly, Zanca et al. [29] reported that in 80% of patients, the scanning area was unnecessarily extended without clinical justification, resulting in an increase in the ED for abdominal scans from 7.9 to 8.4 mSv. Liao et al. [28] further demonstrated that 99% of scans covered an excessively large area, with an average extension of 43.2 mm, leading to an increase in radiation dose by up to 10.38% [28].

In our study, 53.8% of scans had an extended scanning range, while 46.7% had a reduced range. These findings differ from those reported in the previously cited studies. The likely explanation for this discrepancy may be the ongoing, periodic training sessions, which aim to raise awareness among radiographers about the critical role they play in effectively minimizing radiation risks to patients.

The dimensions of the patient in the transverse plane (LAT, AP, and Deff) significantly influence the actual radiation dose absorbed by the body during scanning. Calculating the dose for the entire scan by multiplying measurements from the largest cross-sectional area leads to an unjustified overestimation of the dose. This error arises from the assumption that the scanned area is a cylinder with a constant diameter in the axial section. However, no region of the human body being scanned is a perfect cylinder, and as a result, the diameter varies across different cross-sections.

A potential solution to this issue could be calculating the Deff of the scanned area as the average of the largest and smallest dimensions. However, this approach only provides an approximate value. The most accurate method appears to be estimating the dose based on the Deff for each individual cross-section and summing these values. Unfortunately, this method is highly labor-intensive and impractical for manual measurements in the daily workflow of a CT room. Currently, determining the Deff primarily relies on advanced automated solutions, where artificial intelligence (AI) plays a pivotal role. By analyzing density differences between the patient’s body and air, AI-powered algorithms enable quick, precise, and fully automated assessments of cross-sectional dimensions [30].

In our study, the average Deff of the abdomen was comparable between sexes, with a value of 290.29 mm for men and 290.63 mm for women. Furthermore, in 73.3% of the examinations, the Deff was smaller than the diameter of the reference phantom (320 mm), while in 26.7% of patients, it exceeded this value.

A strong correlation between the Deff and BMI was reported by Boos et al. [31], who analyzed 205 CT abdominal examinations. They demonstrated a high correlation between the Deff and BMI (r = 0.89), as well as between the Deff and body weight (r = 0.84) [31].

Similarly, O’Neill et al. [32] demonstrated a strong correlation (r = 0.88) between the Deff and BMI. They also proposed a linear regression formula for estimating the Deff based on a patient’s BMI [32]:

(7) Deff=0.79×BMI+9.4

In our study, a significant correlation was also found between the Deff and BMI for the entire study group, with a correlation coefficient of r = 0.78.

Currently, available scanners equipped with attenuation-based tube current modulation systems dynamically adjust the tube current based on the patient’s anatomical characteristics. As a result, the tube current increases logarithmically as the patient’s dimensions increase, directly leading to higher radiation doses in patients with larger body mass. This is due to both the automatic increase in tube current and the reduced distance between the patient’s body and the focal point of the tube [33,34,35,36]. To address this, it is essential to use correction parameters that enable precise estimation of the radiation dose received by an individual patient. One such parameter is the SSDE, calculated as the product of the CTDIvol and a conversion factor k (see Formula (3)). This factor is determined based on selected anthropometric characteristics, with the Deff being the most practical indicator [37,38]. According to the guidelines of AAPM Report No. 220, the average dose received by the patient should be estimated using information about the actual attenuation of radiation by tissues [39]. This method involves determining the dose based on the diameter of a water cylinder that attenuates radiation in the same manner as the patient’s body.

However, several publications suggest that there are only minor differences between SSDE estimations based on geometric measurements and those based on more accurate water-equivalent measurements [40,41]. It is important to note, however, that SSDE reflects the dose for a single cross-sectional layer rather than the entire body, making it subject to significant methodological error. For the purposes of this study, the SSDE was calculated using a correction factor determined from the Deff of the maximum cross-sectional area.

CTDIvol represents the average radiation dose for a standard polymethyl methacrylate phantom (320 mm in diameter for abdominal CT) and depends on factors such as the radiation spectrum, tube current, and exposure time. This index facilitates a direct comparison of radiation doses for a single scan layer under different tube parameter settings [42]. However, it does not account for the actual size of the patient or the extent of radiation absorption by their tissues.

CTDIvol can be considered a reliable indicator of a patient’s average dose only if the scanned area’s diameter closely matches the reference phantom diameter. In our study, the mean Deff of patients was 290.44 ± 43.2 mm, deviating from the reference value (320 mm) by an average of 8%. Furthermore, the range of Deff in the study group spanned from 210.14 to 410.12 mm (see Table 3). For a patient with the smallest Deff, the CTDIvol value was underestimated by nearly 34%. Conversely, for a patient with the largest Deff, the CTDIvol value was overestimated by almost 29%. This discrepancy means that the patient with the smallest Deff actually received a higher CTDIvol dose than what was reported in the scan protocol.

The parameter that determines the radiation dose for the entire scan is the DLP, which is calculated as the product of CTDIvol and the length of the scanned area. However, it is important to note that like CTDIvol, the DLP does not account for the patient’s anthropometric differences. As a result, DLP only accurately reflects the actual dose when the diameter of the scanned area matches the diameter of the reference phantom.

In the analyzed patient group, the DLP values for both topographical and diagnostic scans showed statistically significant differences. For abdominal and pelvic scans—areas involving solid organs that absorb more radiation—higher DLP values were observed both for the entire group and separately for women and men. Additionally, the longer scanning length required for abdominal CT directly contributed to the higher DLP values. When comparing scans performed on women and men, DLP values were statistically higher in men. These differences can be attributed to men’s generally larger size (weight and height). However, no differences were observed in the transverse dimensions of the abdominal cavity between the sexes. Thus, the higher DLP values in men were primarily influenced by the increased length of the scanned area, which corresponds to men’s generally greater height.

CTDIvol and DLP are widely used and legally approved indicators for assessing radiation dose during CT scans. Current regulations mandate that these parameters be reported in every scan protocol, making them readily accessible and verifiable within quality control procedures. However, both indicators have significant limitations. Their calculations are based on the diameter of a standard reference phantom, whereas the Deff of a patient’s cross-section rarely matches this value. In our study, nearly 70% of patients had a Deff smaller than the reference value, while over 30% had a larger Deff. These discrepancies between the patient’s Deff and the reference phantom’s diameter result in differences between the actual radiation dose and the calculated values, highlighting the need for more individualized dose estimation methods.

The ED quantifies the extent of exposure and the potential biological damage caused by ionizing radiation. This indicator, as defined by Formula number (4), is the product of the DLP and a coefficient *f* derived from AAPM Report No. 96 [21]. As noted earlier, DLP is a dose parameter calculated for a reference phantom, not for the specific patient. Consequently, the ED represents the radiation dose that a patient, whose dimensions match the reference phantom’s diameter, would receive under a given examination protocol. Despite these limitations, calculating the ED based on DLP is considered reliable, with differences between calculated and actual doses generally not exceeding 15% [21,43,44].

Smith-Bindman et al. [45] reported that the average ED for abdominal CT scans was 10 mSv, ranging from 6 to 16 mSv. By contrast, Li et al. [46] observed significantly lower EDs, averaging around 5.8 mSv in their study. Similarly, an analysis of more than 2 million CT examinations conducted across 151 institutions in 7 countries showed that the EDs for abdominal and pelvic CT scans ranged from 7.0 to 25.7 mSv. Dose differences between countries and institutions reached as high as 30% [47]. These discrepancies were only marginally related to variations in patient size, with factors such as age or the type of scanner having minimal impact. The largest differences in dose parameters were attributed to variations in scanner usage, technical settings, examination protocols, and the preferences of radiographers performing the scans. As a result, it is recommended that reference dose levels be established for individual countries, regions, or even specific CT departments. These reference levels should consider the local population’s characteristics, the type of equipment used, and the diagnostic goals and expectations of the staff [47,48,49].

In our study, the mean ED was 7.72 ± 2.73 mSv for the entire group, with 7.62 ± 2.58 mSv for women and 7.81 ± 2.86 mSv for men. These results are comparable to those reported in other studies.

As previously mentioned, calculating SSDE without considering its role in determining radiation dose parameters for the entire study can be regarded as a methodological error. Therefore, in this work, an attempt was made to calculate the corresponding values of the DLP and ED radiation doses based on parameters corrected for the patient’s actual dimensions. The proposed new parameters, DLPss and EDss, were calculated using formulas analogous to the traditional parameters (see Section 2), but with the inclusion of correction factors accounting for the patient’s actual size (see Formula numbers (5) and (6)).

Our results clearly demonstrate that the DLPss and EDss values, obtained through mathematical adjustments, deviated significantly from those calculated using traditional methods. Specifically, for chest CT, the DLPss was on average 20% higher, while the EDss was 19% higher. Similarly, for abdominal and pelvic CT, these values were higher by 21% and 22%, respectively. These findings highlight that using such modified parameters enables a more accurate estimation of radiation doses, accounting for the varying body structures of patients. This is crucial for precise examination planning and for assessing the actual radiation risk associated with specific diagnostic procedures. The significant discrepancies observed between the traditional indices and the proposed parameters strongly indicate the need for updating the widely used dose assessment standards.

The use of AI in patient radiation protection is becoming increasingly prevalent, as demonstrated by the development of advanced algorithms designed to automatically determine optimal radiation doses or reconstruct high-quality images [50,51,52]. These AI algorithms consider various parameters, including patient dimensions, age, and specific diagnostic requirements. Studies indicate that such solutions can reduce radiation doses by up to 70% without compromising diagnostic effectiveness [53,54]. Furthermore, permanent access to data stored on servers enables the continuous optimization of research procedures with minimal changes to existing infrastructure. AI is poised to become a key tool for analyzing patient data and predicting individual susceptibility to radiation effects. This capability will facilitate more informed decisions regarding the necessity and frequency of CT scans. Such an integrated approach not only enhances diagnostic effectiveness and safety but also ensures the highest standard of patient care.

Our study has several limitations. First, determining the proposed new parameters (DLPss and EDss) is time-consuming and challenging to implement in daily practice. The use of AI algorithms could likely streamline and simplify this process. Second, the study was conducted in a single institution, which may limit the generalizability of the results to facilities with different scanner types and varying research protocols. Multicenter studies would likely enhance the validation of these findings. Third, the lack of long-term data precludes an assessment of the clinical impact of DLPss and EDss in radiation dose estimation, particularly regarding patient treatment outcomes or potential complications caused by ionizing radiation. Finally, while the introduction of these new dose metrics is innovative, it is critical to investigate how changes in scanning parameters or variations in the patient’s condition may influence their accuracy.

## 5. Conclusions and Future Scope

Traditional methods of dose assessment, such as DLP and ED, have notable limitations, primarily due to their inability to account for individual patient anthropometric characteristics. The new parameters we propose for dose estimation—DLPss and EDss—address this gap and have the potential to revolutionize the way radiation doses are assessed in radiology. While the initial results are promising, the widespread implementation of these new metrics presents challenges. Overcoming these obstacles will require not only technological advancements but also changes in the approach of clinicians and the training of medical personnel. Future studies should focus on validating these proposed indicators in diverse patient populations and clinical settings. Moreover, developing AI tools for the automatic calibration and optimization of DLPss and EDss could significantly accelerate their integration into routine clinical practice.

## Figures and Tables

**Table 1:** Anthropometric parameters of the study group.

	Mean	Female	Male	*p*
Age (years)	59.65 ± 13.82	65.89 ± 12.91	63.15 ± 13.15	*p* = 0.024
Weight (kg)	74.88 ± 16.46	69.19 ± 13.24	79.67 ± 17.40	*p* < 0.05
Height (cm)	170.04 ± 9.84	162.06 ± 6.33	176.77 ± 6.77	*p* < 0.05
BMI (kg/m^2^)	25.92 ± 5.34	26.42 ± 5.24	25.49 ± 5.40	*p* = 0.29

**Table 2:** Topographic and diagnostic scan length.

	Topographic Scan Length (mm)	Diagnostic Scan Length (mm)	Average Length Change (mm)
Female	476.98 ± 44.19	457.45 ± 47.78	−19.53 ± 57.4
Male	493.10 ± 43.93	488.76 ± 66.78	−4.64 ± 68.99
Mean	485.73 ± 44.69	474.28 ± 60.75	−11.45 ± 64.2

**Table 3:** Cross-sectional dimensions of abdominal and pelvic CT (LAT—lateral dimension, AP—anteroposterior dimension, Deff—effective diameter, SD—standard deviation, No.—number of patients).

	Dimension (mm)	No.	Mean	Min	Max	SD
All patients	LAT	247	350.30	270.74	500.65	4.60
	AP	247	240.63	150.91	380.54	4.43
	Deff	247	290.44	210.14	410.12	4.32
Female	LAT	113	350.97	280.00	500.65	4.78
	AP	113	240.48	150.91	350.45	4.07
	Deff	113	290.63	210.87	390.43	4.16
Male	LAT	134	340.73	270.74	470.36	4.38
	AP	134	240.76	150.95	380.54	4.72
	Deff	134	290.29	210.14	410.12	4.47

**Table 4:** Correlation of CTDIvol with selected anthropometric parameters.

CTDIvol	Female	Male
Age	0.314241	0.224551
Weight	0.652626	0.832890
Height	−0.071009	0.127875
BMI	0.644885	0.827138
LAT	0.938334	0.911138
AP	0.783562	0.865463
Deff	0.852242	0.915642
Scan length	0.265650	0.405267

**Table 5:** Comparison of DLP and DLPss (mGy·cm) values between chest and abdominal/pelvic CT.

Region of Examination	Mean	Female	Male	*p*
Chest CT (DPL)	289.51 ± 99.82	258.88 ± 98.97	314.98 ± 93.11	*p* < 0.05
Chest CT (DPLss)	349.9 ± 81.66	323.53 ± 78.69	364.89 ± 79.87	*p* < 0.05
Abdominal CT (DLP)	514.88 ± 195.24	508.35 ± 171.73	520.38 ± 190.41	*p* = 0.69
Abdominal CT (DLPss)	627.83 ± 145.32	617.47 ± 136.87	636.57± 152.03	*p* = 0.166

**Table 6:** Comparison of ED and EDss (mSv) values between chest and abdominal CT.

Region of Examination	Mean	Female	Male	*p*
Chest CT (ED)	4.01 ± 1.4	3.66 ± 1.39	4.41 ± 1.3	*p* < 0.05
Chest CT (EDss)	4.81 ± 1.14	4.53 ± 1.1	5.11 ± 1.12	*p* < 0.001
Abdominal CT (ED)	7.72 ± 2.73	7.62 ± 2.58	7.81 ± 2.86	*p* = 0.69
Abdominal CT (EDss)	9.42 ± 2.18	9.25 ± 2.06	9.55 ± 2.28	*p* = 0.16

## Data Availability

The datasets used and analyzed during the current study are available from the corresponding author upon reasonable request.

## Abbreviations

The following abbreviations are used in this manuscript:

CT	computed tomography
DLP	dose–length product
ED	effective dose
SSDE	size-specific dose estimate
EDss	size-specific effective dose
DLPss	size-specific dose–length product
BMI	body mass index
Deff	effective diameter
CTDIvol	volume computed tomography dose index
LAT	lateral
AP	anterior–posterior
SD	standard deviation
CNR	contrast-to-noise ratio
ALARA	as low as reasonably achievable
ALARP	as low as reasonably practicable
AI	artificial intelligence
ATCM	attenuation-based tube current modulation
AAPM	American Association of Physicists in Medicine
